# Baseline Clinical and Laboratory Predictors of Treatment Requirement in Chronic Lymphocytic Leukemia: A Retrospective Cohort Study Using Hierarchical Modeling

**DOI:** 10.3390/diagnostics16132003

**Published:** 2026-06-26

**Authors:** Hasan Göze, Birgül Öneç

**Affiliations:** 1 Division of Hematology, Department of Internal Medicine, University of Health Sciences, Istanbul Basaksehir Cam and Sakura City Hospital, İstanbul 34000, Türkiye; 2 Division of Hematology, Department of Internal Medicine, Faculty of Medicine, Duzce University, Duzce 81100, Türkiye

**Keywords:** chronic lymphocytic leukemia, risk stratification, treatment requirement, prognostic factors

## Abstract

**Background/Objectives**: Chronic lymphocytic leukemia (CLL) is characterized by a highly heterogeneous clinical course, with some patients remaining stable for years while others require early treatment. Identifying reliable and easily accessible predictors of treatment requirement at diagnosis remains an important clinical challenge. **Methods:** This retrospective cohort study included 226 patients diagnosed with CLL between 2015 and 2024 at a tertiary care center. Baseline demographic, clinical, and laboratory parameters were analyzed. Univariate and multivariable logistic regression analyses were performed to identify independent predictors of treatment requirement. A hierarchical mixed-effects model was constructed to account for temporal clustering across diagnostic periods. A clinical risk score was derived from independent predictors, using regression coefficient-based weighting, and its discriminative performance was evaluated using receiver operating characteristic (ROC) analysis. **Results**: A total of 226 patients were included (mean age: 62.4 ± 13.8 years; 56.6% male). During follow-up, 104 patients (46.0%) required treatment. Lower hemoglobin and platelet levels, higher lymphocyte counts and LDH levels, and the presence of B symptoms, splenomegaly, and advanced disease stage were independently associated with treatment requirement. These associations remained significant in hierarchical modeling. The derived risk score demonstrated acceptable discriminative ability (AUC: 0.84; 95% CI: 0.79–0.89), with a cut-off value of ≥4 yielding a sensitivity of 81.7% and specificity of 73.8%. **Conclusions**: Baseline clinical and laboratory parameters are associated with treatment requirement in CLL. A combination of readily available variables may support risk stratification at diagnosis. The proposed risk score may provide a practical adjunct to routine clinical assessment, particularly in settings where advanced molecular testing is not readily available; however, external validation in independent cohorts is required before clinical implementation.

## 1. Introduction

Chronic lymphocytic leukemia (CLL) is the most common adult leukemia in Western populations and is characterized by a highly heterogeneous clinical course. While some patients remain asymptomatic for many years without requiring treatment, others experience rapid disease progression and early need for therapy. This marked heterogeneity underscores the importance of accurate risk stratification at diagnosis and the development of individualized follow-up and management strategies [1,2].

Traditionally, staging systems such as Rai and Binet have been widely used to assess disease burden and prognosis in CLL [3]. However, these systems are primarily based on clinical findings and may not fully capture the biological behavior of the disease or reliably predict treatment requirement. Consequently, there has been increasing interest in identifying additional prognostic markers, particularly readily available laboratory parameters. Variables such as hemoglobin level, platelet count, lymphocyte count, and lactate dehydrogenase (LDH) have been reported to be associated with disease activity and clinical outcomes [4,5,6,7]. In addition to these routinely available parameters, molecular and cytogenetic markers such as IGHV mutational status and TP53 abnormalities have been shown to significantly influence disease progression, treatment response, and survival in patients with CLL [5,6,7,8,9,10].

Despite these advances, several validated prognostic models, including the Chronic Lymphocytic Leukemia International Prognostic Index (CLL-IPI) and the International Prognostic Score for Early-stage CLL (IPS-E), have been developed to improve risk stratification in CLL [8,9,10]. However, these models often require molecular and cytogenetic information that may not be universally available in routine clinical practice. Therefore, the evaluation of readily available clinical and laboratory parameters remains clinically relevant, particularly in settings with limited access to advanced diagnostic testing. Furthermore, temporal variations in clinical practice and patient characteristics over time may influence outcomes, highlighting the need for analytical approaches that account for such clustering effects. Although hierarchical and mixed-effects models have increasingly been used in biomedical research to address clustered data structures, their application in studies evaluating treatment requirement in CLL remains relatively limited [11,12].

The present study aims to address these gaps by comprehensively evaluating baseline clinical and laboratory parameters to predict treatment requirement in patients with CLL. A hierarchical mixed-effects regression model was employed to account for potential temporal clustering, and a clinically applicable risk score was constructed based on independently significant predictors. The diagnostic performance of this risk score was further assessed using receiver operating characteristic (ROC) analysis.

The primary objective of this study was to investigate the role of baseline clinical and laboratory variables in predicting treatment requirement in patients with CLL and to develop a practical risk scoring system for clinical use. Rather than replacing established prognostic systems, the proposed model was designed as a complementary approach based on routinely available clinical information that may support risk assessment when comprehensive molecular profiling is unavailable. We hypothesized that a combination of readily available clinical and laboratory parameters could provide significant predictive value for treatment requirement and serve as a complementary tool to existing staging systems.

## 2. Materials and Methods

### 2.1. Study Design, Setting, and Ethics

This study was designed as a retrospective observational cohort study conducted at a tertiary care university hospital, which serves as a regional referral center for hematologic diseases. The study included patients diagnosed with CLL over a 10-year period between January 2015 and December 2024. All data were obtained from the hospital’s electronic medical records system and reviewed retrospectively.

The study was conducted in accordance with the principles of the Declaration of Helsinki and adhered to the Strengthening the Reporting of Observational Studies in Epidemiology (STROBE) guidelines. Ethical approval was obtained from the Düzce University Non-Interventional Clinical Research Ethics Committee (approval number: 2025/98; date: 7 April 2025). Due to the retrospective nature of the study, the requirement for informed consent was waived by the ethics committee.

### 2.2. Study Population

Patients were identified through the hospital’s electronic medical records system based on diagnostic coding for chronic lymphocytic leukemia (CLL). During the study period, a total of 312 patients diagnosed with CLL were initially screened. After applying the predefined inclusion and exclusion criteria, 226 consecutive adult patients (≥18 years) were included in the final analysis.

Inclusion criteria were defined as a confirmed diagnosis of CLL based on standard clinical, laboratory, and immunophenotypic findings and availability of complete baseline clinical and laboratory data at the time of diagnosis. Patients with insufficient medical records or missing key variables required for the primary analyses were excluded.

Exclusion criteria included patients with incomplete follow-up data, those with concomitant hematologic malignancies other than CLL, and patients with repeated or duplicate records. In cases where multiple entries were identified for the same patient, only the initial diagnostic record was included to avoid duplication bias.

**Figure 1:**
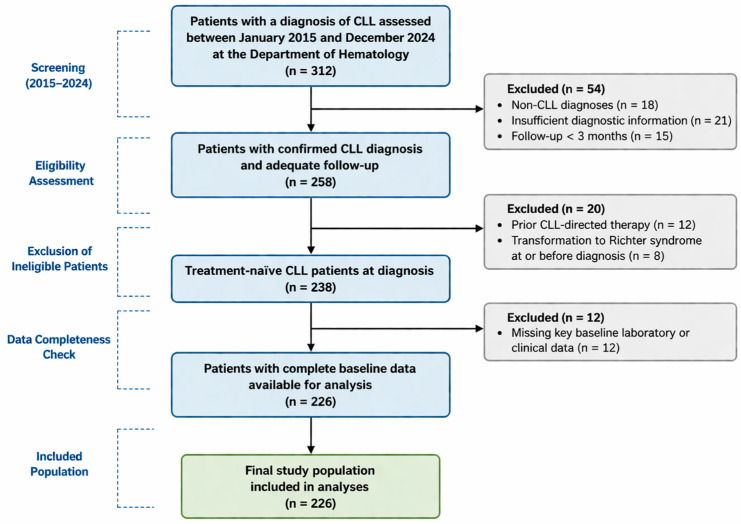
Flow diagram of patient selection and inclusion process.

The patient selection process is summarized in Figure 1.

### 2.3. Data Collection and Variables

Clinical information was obtained through a retrospective review of the institutional electronic health record database using a predefined data abstraction protocol. Variables of interest were collected at diagnosis and throughout the follow-up period. Patient characteristics included age and sex. Disease-related clinical findings comprised B symptoms, including fever, night sweats, and unexplained weight loss, as well as splenomegaly, hepatomegaly, and lymphadenopathy documented during routine clinical assessment and radiological evaluation. Direct antiglobulin (Coombs) test results were recorded when available in the medical records. Baseline laboratory assessments performed at diagnosis included hemoglobin concentration, total white blood cell count, absolute lymphocyte count, and platelet count. Hematological analyses were conducted using automated laboratory systems routinely employed in the hospital. Biochemical markers, including lactate dehydrogenase and beta-2 microglobulin, were measured according to standard laboratory procedures. To ensure comparability across patients, only values obtained before initiation of CLL-specific treatment were considered. All laboratory analyses were performed within the same institutional laboratory as part of routine patient care. Disease severity at presentation was classified according to the Rai staging system. For analytical purposes, patients were grouped into low-risk (stage 0), intermediate-risk (stages I–II), and high-risk (stages III–IV) categories. Outcome measures included treatment requirement, disease progression, and all-cause mortality during follow-up. The primary outcome was treatment requirement, defined as initiation of any therapy directed toward chronic lymphocytic leukemia after diagnosis. Disease progression was identified based on documented clinical, laboratory, or imaging findings indicating worsening disease status according to accepted CLL evaluation criteria. Follow-up time was calculated from the date of diagnosis until the most recent clinical assessment or death, whichever occurred first.

### 2.4. Missing Data

Data completeness was evaluated before statistical analyses were undertaken. Patients lacking essential baseline clinical or laboratory information necessary for the primary analyses were excluded during cohort assembly. Consequently, only individuals with complete data for the variables of interest were retained in the final dataset. The frequency of missing observations among eligible variables was minimal. Because no evidence of a systematic missing-data mechanism was observed and the overall proportion of missing values was low, missing-data imputation techniques were not implemented. Analyses were therefore conducted using a complete-case approach, which was considered appropriate for the study design and available dataset.

### 2.5. Statistical Analysis

Data analyses were conducted using IBM SPSS Statistics version 26.0 (IBM Corp., Armonk, NY, USA). Distributional assumptions for continuous variables were evaluated through graphical inspection and the Shapiro–Wilk test. Variables showing a normal distribution are reported as mean ± standard deviation, whereas skewed variables are presented as median with interquartile range. Descriptive results for categorical variables are expressed as frequencies and percentages.

Between-group comparisons were performed according to data distribution. The independent-samples *t* test was applied for normally distributed continuous variables, while the Mann–Whitney U test was used when normality assumptions were not met. Comparisons involving more than two groups were analyzed using one-way analysis of variance or the Kruskal–Wallis test, as appropriate. Associations between categorical variables were examined using the chi-square test or Fisher’s exact test when required.

Potential predictors of treatment requirement were initially explored using univariate logistic regression analyses. Variables demonstrating statistical significance and those considered clinically important were subsequently entered into a multivariable logistic regression model to identify factors independently associated with treatment initiation during follow-up. Multicollinearity was evaluated before model construction using correlation matrices and variance inflation factors. Because beta-2 microglobulin showed substantial overlap with other markers reflecting disease burden, it was not included in the final multivariable model. Effect estimates are presented as odds ratios with corresponding 95% confidence intervals.

To evaluate potential temporal variation across the study period, patients were classified into three diagnostic intervals (2015–2017, 2018–2020, and 2021–2024). A mixed-effects logistic regression model including a random intercept for diagnostic period was then developed. This approach was selected to account for possible differences in clinical practice patterns over time. Demographic characteristics, laboratory findings, and disease-related variables were entered as fixed effects. Model performance was summarized using random-effect variance, intraclass correlation coefficient, Akaike information criterion, and Bayesian information criterion values.

The predictive performance of laboratory parameters and the derived clinical risk score was assessed using receiver operating characteristic curve analysis. Area under the curve values with 95% confidence intervals were calculated together with sensitivity, specificity, and optimal threshold values. Optimal cut-offs were identified according to the Youden index. A clinical risk score was generated from the independent predictors retained in the multivariable model, with individual point allocations reflecting the relative magnitude of regression coefficients. The resulting total score was used to stratify patients into low-, intermediate-, and high-risk categories. Since no external validation dataset was available, the proposed score should be regarded as preliminary and requires independent validation in future studies.

Statistical significance was defined as a two-sided *p*-value below 0.05. Sample size adequacy was evaluated using G*Power version 3.1.9.7 (Heinrich Heine University Düsseldorf, Düsseldorf, Germany). Based on assumptions derived from a previously published logistic regression study, the minimum required sample size was estimated to be 184 participants. The inclusion of 226 patients, therefore, provided sufficient statistical power for the planned analyses [7].

## 3. Results

Baseline demographic, clinical, and laboratory characteristics of the study cohort are presented in Table 1. A total of 226 patients were included in the analysis. The mean age was 62.4 ± 13.8 years, and 128 patients (56.6%) were male. B symptoms were present in 64 patients (28.3%), while splenomegaly and lymphadenopathy were observed in 72 (31.9%) and 118 (52.2%) patients, respectively. The mean hemoglobin level was 11.2 ± 2.1 g/dL, the mean white blood cell count was 18.6 ± 12.4 × 10^9^/L, and the mean absolute lymphocyte count was 13.2 ± 10.8 × 10^9^/L. The mean platelet count was 168 ± 82 × 10^9^/L. Median LDH level was 410 U/L (IQR: 290–620), and median beta-2 microglobulin level was 3.4 mg/L (IQR: 2.1–5.8). According to disease staging, 98 patients (43.4%) were classified as low stage, 76 (33.6%) as intermediate stage, and 52 (23.0%) as high stage. During follow-up, 104 patients (46.0%) required treatment, 88 (38.9%) experienced progression, and 42 (18.6%) died. Median follow-up duration was 38 months (IQR: 18–64).

Comparisons according to disease stage are presented in Table 2. Advancing disease stage was associated with older age, lower hemoglobin and platelet levels, and higher white blood cell and absolute lymphocyte counts (all *p* < 0.05). Similarly, the prevalence of B symptoms, splenomegaly, and treatment requirement increased significantly across disease stages (all *p* < 0.001).

Patients requiring treatment exhibited a less favorable clinical and laboratory profile compared with those who remained untreated (Table 3). Treatment requirement was associated with older age, lower hemoglobin and platelet levels, higher lymphocyte counts and LDH levels, and a greater prevalence of B symptoms, splenomegaly, and high-stage disease (all *p* < 0.001).

Similarly, patients who experienced disease progression demonstrated significantly older age, more advanced disease characteristics, lower hemoglobin and platelet levels, and higher lymphocyte and LDH levels compared with patients without progression (Table 4). B symptoms, splenomegaly, high-stage disease, and treatment requirement were also significantly more frequent among patients with progression (all *p* < 0.001).

Univariate logistic regression analysis for predicting treatment requirement is presented in Table 5. Increasing age was associated with higher odds of treatment requirement (OR: 1.04, 95% CI: 1.02–1.06, *p* < 0.001). Lower hemoglobin levels (OR: 0.72, 95% CI: 0.64–0.81, *p* < 0.001) and lower platelet counts (OR: 0.98, 95% CI: 0.97–0.99, *p* < 0.001) were significantly associated with treatment requirement.

Higher white blood cell counts (OR: 1.05, 95% CI: 1.03–1.07, *p* < 0.001) and higher absolute lymphocyte counts (OR: 1.06, 95% CI: 1.03–1.08, *p* < 0.001) were also associated with increased likelihood of treatment requirement. Elevated LDH levels were significantly associated with treatment requirement (OR: 1.22 per 100 U/L increase, 95% CI: 1.14–1.31, *p* < 0.001).

Among clinical variables, the presence of B symptoms (OR: 4.65, 95% CI: 2.50–8.66, *p* < 0.001), splenomegaly (OR: 2.95, 95% CI: 1.65–5.28, *p* < 0.001), and high-stage disease (OR: 5.78, 95% CI: 2.90–11.52, *p* < 0.001) were strongly associated with treatment requirement.

Multivariable logistic regression analysis for predicting treatment requirement is presented in Table 6. Increasing age remained independently associated with treatment requirement (adjusted OR: 1.03, 95% CI: 1.01–1.05, *p* = 0.004). Lower hemoglobin levels were significantly associated with treatment requirement (adjusted OR: 0.78, 95% CI: 0.69–0.89, *p* < 0.001). Higher absolute lymphocyte counts (adjusted OR: 1.04, 95% CI: 1.02–1.07, *p* < 0.001) and elevated LDH levels (adjusted OR: 1.16 per 100 U/L increase, 95% CI: 1.08–1.25, *p* < 0.001) remained significant independent predictors. Platelet count also remained independently associated with treatment requirement (adjusted OR: 0.99, 95% CI: 0.98–0.99, *p* = 0.002). Among clinical variables, the presence of B symptoms (adjusted OR: 3.12, 95% CI: 1.55–6.28, *p* = 0.001), splenomegaly (adjusted OR: 2.21, 95% CI: 1.18–4.14, *p* = 0.013), and high-stage disease (adjusted OR: 3.85, 95% CI: 1.85–8.02, *p* < 0.001) remained independently associated with treatment requirement. Assessment of multicollinearity demonstrated no significant collinearity among variables retained in the final multivariable model (all VIF values < 3.0). Beta-2 microglobulin showed substantial correlation with other disease burden indicators and was therefore excluded from the final model.

Hierarchical mixed-effects logistic regression analysis for predicting treatment requirement, accounting for clustering by diagnostic period, is presented in Table 7. Increasing age remained independently associated with treatment requirement (adjusted OR: 1.03, 95% CI: 1.01–1.05, *p* = 0.006). Lower hemoglobin levels (adjusted OR: 0.79, 95% CI: 0.70–0.90, *p* < 0.001), higher absolute lymphocyte counts (adjusted OR: 1.04, 95% CI: 1.02–1.07, *p* < 0.001), and elevated LDH levels (adjusted OR: 1.15 per 100 U/L increase, 95% CI: 1.07–1.24, *p* < 0.001) remained significant predictors. Platelet count (adjusted OR: 0.99, 95% CI: 0.98–0.99, *p* = 0.003), B symptoms (adjusted OR: 2.98, 95% CI: 1.48–6.02, *p* = 0.002), splenomegaly (adjusted OR: 2.10, 95% CI: 1.12–3.95, *p* = 0.021), and high-stage disease (adjusted OR: 3.62, 95% CI: 1.72–7.63, *p* < 0.001) also remained independently associated with treatment requirement.

Random effects and model fit statistics of the hierarchical model are presented in Table 8. The variance of the random intercept for the diagnostic period was 0.18, corresponding to an intraclass correlation coefficient of 0.05, indicating a modest clustering effect. Model fit statistics included an AIC of 214.6 and a BIC of 238.9.

Receiver operating characteristic (ROC) analysis for laboratory parameters and the clinical risk score is presented in Table 9. The area under the curve (AUC) for hemoglobin was 0.74 (95% CI: 0.68–0.80), for absolute lymphocyte count was 0.71 (95% CI: 0.65–0.77), and for platelet count was 0.69 (95% CI: 0.63–0.75). LDH demonstrated a higher discriminative performance with an AUC of 0.78 (95% CI: 0.72–0.83). The clinical risk score showed the highest discriminative ability with an AUC of 0.84 (95% CI: 0.79–0.89). A cut-off value of ≥4 points yielded a sensitivity of 81.7% and specificity of 73.8% for predicting treatment requirement. The discriminative performance of the clinical risk score was superior to that of individual laboratory parameters, supporting the incremental value of combining multiple predictors into a single risk stratification tool.

The distribution of treatment requirement and clinical outcomes according to risk score categories is presented in Table 10. Among patients in the low-risk group, 12 (15.0%) required treatment, compared with 42 (45.7%) in the intermediate-risk group and 50 (92.6%) in the high-risk group (*p* < 0.001). Disease progression was observed in 10 patients (12.5%) in the low-risk group, 34 (37.0%) in the intermediate-risk group, and 44 (81.5%) in the high-risk group (*p* < 0.001). Mortality rates were 4 (5.0%), 14 (15.2%), and 24 (44.4%) in the low-, intermediate-, and high-risk groups, respectively (*p* < 0.001).

## 4. Discussion

The present study demonstrates that baseline clinical and laboratory parameters are significantly associated with treatment requirement in patients with CLL. In particular, age, hemoglobin level, lymphocyte count, platelet count, LDH, B symptoms, splenomegaly, and disease stage were identified as key determinants of treatment initiation. These findings support the concept that routinely available clinical and laboratory data can provide meaningful prognostic information at the time of diagnosis.

Although traditional staging systems remain fundamental in the evaluation of CLL, our results suggest that their predictive capacity may be enhanced when combined with additional laboratory parameters. The observed associations between disease burden indicators and treatment requirement are consistent with the known biological heterogeneity of CLL, where both tumor load and host-related factors contribute to disease progression. From a clinical perspective, this integrated approach may facilitate more refined risk stratification and support early identification of patients who are more likely to require treatment [13,14,15]. Furthermore, contemporary prognostic systems such as the Chronic Lymphocytic Leukemia International Prognostic Index (CLL-IPI) and the International Prognostic Score for Early-stage CLL (IPS-E) have demonstrated substantial value in predicting disease outcomes through the incorporation of clinical, laboratory, molecular, and cytogenetic variables. Nevertheless, the application of these models may be limited in some settings due to the requirement for specialized testing, highlighting the continuing relevance of routinely available parameters in risk assessment [14,15]. One of the notable methodological aspects of the present study is the use of a hierarchical mixed-effects model to account for potential temporal clustering across diagnostic periods. Although the magnitude of clustering, as reflected by the intraclass correlation coefficient, was modest, incorporating this structure allowed for a more robust estimation of independent predictors by accounting for potential variations in clinical practice and patient characteristics over time. In clinical settings, diagnostic approaches, laboratory standardization, and treatment thresholds may evolve, potentially influencing observed outcomes [16,17].

While the observed clustering effect was limited, its inclusion strengthens the methodological rigor of the analysis and reduces the risk of biased estimates that may arise from ignoring time-related dependencies. Previous studies in CLL have largely relied on conventional regression models without considering such hierarchical structures, which may overlook subtle but relevant sources of variability. Therefore, the application of a mixed-effects framework in the present study may provide a more nuanced understanding of predictors of treatment requirement. However, given the relatively low degree of clustering observed, these findings should be interpreted with caution, and further studies are needed to clarify the clinical relevance of temporal effects in CLL cohorts. The present study identified both laboratory and clinical variables as independent predictors of treatment requirement. Lower hemoglobin levels and reduced platelet counts were associated with an increased likelihood of treatment initiation, consistent with the well-established role of cytopenias as indicators of advanced disease burden and bone marrow involvement in CLL [18,19]. These findings remained significant after adjustment for other variables, supporting the notion that cytopenias may contribute additional prognostic information beyond conventional staging systems. However, cytopenias in CLL may also arise from alternative mechanisms, including autoimmune complications, and should therefore be interpreted within the broader clinical context [20,21]. Similarly, elevated LDH levels and higher absolute lymphocyte counts were independently associated with treatment requirement. LDH is widely regarded as a marker of increased cellular turnover and tumor burden, whereas lymphocyte count directly reflects circulating disease burden [22,23,24]. The persistence of these variables in both multivariable and hierarchical models suggests that they provide prognostic information beyond traditional staging systems. Previous studies have likewise demonstrated associations between elevated lymphocyte counts, increased disease activity, and adverse clinical outcomes [25,26,27]. Nevertheless, LDH may be influenced by non-malignant conditions, and lymphocyte counts may fluctuate over time, emphasizing the need for cautious interpretation within the overall clinical context [28].

Clinical findings, including the presence of B symptoms, splenomegaly, and advanced disease stage, were also strongly associated with treatment requirement in this cohort. These parameters reflect the clinical burden of disease and have long been recognized as key components in the assessment of disease activity in CLL. The association between B symptoms and treatment initiation is particularly expected, as systemic symptoms such as fever, night sweats, and weight loss are often indicative of biologically active or progressive disease [15,29]. Similarly, splenomegaly may reflect increased tumor infiltration and disease dissemination, contributing to both symptomatic burden and hematologic compromise. In line with previous literature, our findings demonstrate that patients with splenomegaly are more likely to require treatment, supporting its role as a clinically meaningful marker of disease progression. Furthermore, disease stage remained one of the strongest predictors in both multivariable and hierarchical models, reinforcing the continued relevance of traditional staging systems in clinical decision-making [30,31]. However, while these clinical variables provide important information, they may not fully capture the underlying biological heterogeneity of CLL. The persistence of their predictive value alongside laboratory parameters in our models suggests that a combined approach integrating both clinical and laboratory features may offer a more comprehensive assessment of disease behavior. Nevertheless, these findings should be interpreted cautiously, as clinical features may be subject to interobserver variability and may evolve over time [1,32]. A secondary finding of the present study is the derivation of a clinically applicable risk score based on independent predictors identified at diagnosis. The scoring approach demonstrated acceptable discriminative ability in ROC analysis and allowed stratification of patients into risk groups with progressively increasing rates of treatment requirement, disease progression, and mortality. These findings suggest that combining multiple routinely available clinical and laboratory variables may improve risk estimation compared to individual parameters alone.

An important consideration is the relationship of the proposed score to established prognostic systems such as CLL-IPI and IPS-E. These validated models incorporate molecular and cytogenetic variables, including TP53 abnormalities, IGHV mutational status, and β2-microglobulin levels, and have demonstrated substantial prognostic value in CLL [14,25,32]. In contrast, the present score was intentionally developed using routinely available clinical and laboratory parameters. Therefore, it should not be viewed as a replacement for established prognostic systems. Rather, it may serve as a complementary tool in situations where comprehensive molecular profiling is unavailable or incomplete. The acceptable discriminative performance observed in the present study suggests that readily available variables may still provide meaningful prognostic information, although external validation and direct comparison with existing prognostic indices are required.

From a practical standpoint, the findings of the present study may have implications for clinical decision-making in patients with CLL. The identification of readily available baseline predictors of treatment requirement may support more individualized follow-up strategies, particularly in patients classified as higher risk. Although current guidelines emphasize a watch-and-wait approach in early-stage disease, the integration of simple clinical and laboratory parameters may help refine risk assessment and identify patients who may benefit from closer monitoring [25,33]. However, these implications should be interpreted cautiously and require validation in prospective studies before routine clinical implementation.

Several limitations of the present study should be acknowledged. First, the retrospective design inherently introduces the possibility of selection bias, information bias, and residual confounding despite the inclusion of consecutive patients based on predefined criteria. Second, the study was conducted at a single tertiary referral center, which may limit the generalizability of the findings to broader CLL populations, particularly those managed in community-based settings. Third, although a comprehensive set of clinical and laboratory variables was evaluated, important molecular and cytogenetic prognostic markers, including IGHV mutational status, TP53 abnormalities, del(17p), del(13q14), and other genomic alterations, were not systematically available for all patients and therefore could not be incorporated into the analyses. Furthermore, data regarding del(13q14), which is generally considered a favorable prognostic marker in CLL, were not consistently available and therefore could not be evaluated separately. The absence of these established prognostic factors may have influenced risk estimation and limited a direct comparison with validated prognostic systems such as CLL-IPI and IPS-E. Fourth, the proposed risk score was both developed and tested within the same cohort, raising the possibility of overfitting and optimistic performance estimates. Consequently, the score should be considered exploratory and requires external validation in independent cohorts before routine clinical application. In addition, formal internal validation procedures, such as bootstrap resampling or cross-validation, were not performed, and therefore, the robustness of the model should be interpreted with caution. Fifth, although a hierarchical mixed-effects approach was used to account for temporal clustering, the observed clustering effect was modest, suggesting limited temporal variability while not completely excluding residual confounding related to changes in diagnostic and treatment practices over the study period. Finally, several clinical variables, including B symptoms and physical examination findings, may be subject to interobserver variability, and laboratory parameters were assessed only at diagnosis without consideration of longitudinal changes during follow-up. Future prospective multicenter studies incorporating serial measurements, molecular profiling, cytogenetic data, formal model validation procedures, and external validation cohorts are warranted to further refine and validate predictive models for treatment requirement in CLL.

## 5. Conclusions

In conclusion, the findings of this study suggest that several baseline clinical and laboratory parameters, including hemoglobin level, platelet count, lymphocyte count, LDH, B symptoms, splenomegaly, and disease stage, are significantly associated with treatment requirement in patients with chronic lymphocytic leukemia. These readily available variables may provide meaningful prognostic information at the time of diagnosis and may complement existing risk assessment strategies in routine clinical practice. The consistency of findings across conventional and hierarchical regression models supports the robustness of the identified associations. Furthermore, the proposed risk score demonstrated acceptable discriminative performance and successfully stratified patients according to their likelihood of treatment requirement, disease progression, and mortality. However, the proposed score should not be considered a replacement for established prognostic systems such as CLL-IPI or IPS-E. Rather, it should be regarded as an exploratory and complementary tool based on routinely available clinical information. Given the retrospective single-center design and the lack of external validation, further prospective multicenter studies incorporating molecular and cytogenetic markers are required to confirm the clinical utility, generalizability, and incremental value of the proposed model.

## Figures and Tables

**Table 1:** Baseline demographic, clinical, and laboratory characteristics of the study cohort.

Variable	Total (*n* = 226)
Age (years), mean ± SD	62.4 ± 13.8
Sex (Male), *n* (%)	128 (56.6)
**Clinical Features, *n* (%)**	
B symptoms	64 (28.3)
Splenomegaly	72 (31.9)
Lymphadenopathy	118 (52.2)
**Laboratory Parameters**	
Hemoglobin (g/dL), mean ± SD	11.2 ± 2.1
White blood cells (×10^9^/L), mean ± SD	18.6 ± 12.4
Absolute lymphocyte count (×10^9^/L), mean ± SD	13.2 ± 10.8
Platelet (×10^9^/L), mean ± SD	168 ± 82
LDH (U/L), median (IQR)	410 (290–620)
Beta-2 microglobulin (mg/L), median (IQR)	3.4 (2.1–5.8)
**Disease Stage, *n* (%)**	
Low stage	98 (43.4)
Intermediate stage	76 (33.6)
High stage	52 (23.0)
**Clinical Outcomes, *n* (%)**	
Treatment required	104 (46.0)
Progression	88 (38.9)
Death	42 (18.6)
Follow-up duration (months), median (IQR)	38 (18–64)

SD: Standard deviation; IQR: Interquartile range; LDH: Lactate dehydrogenase.

**Table 2:** Clinical and laboratory characteristics according to disease stage.

Variable	Low Stage (*n* = 98)	Intermediate Stage (*n* = 76)	High Stage (*n* = 52)	*p*-Value
Age (years), mean ± SD	59.8 ± 13.2	63.7 ± 14.1	66.1 ± 13.9	0.01
Hemoglobin (g/dL), mean ± SD	12.4 ± 1.8	11.1 ± 1.9	9.6 ± 2.1	<0.001
White blood cells (×10^9^/L), mean ± SD	14.2 ± 9.8	19.8 ± 11.6	25.6 ± 14.3	<0.001
Absolute lymphocyte count (×10^9^/L), mean ± SD	9.8 ± 7.6	14.5 ± 9.2	20.4 ± 12.6	<0.001
Platelet (×10^9^/L), mean ± SD	198 ± 72	162 ± 80	118 ± 85	<0.001
**Clinical Findings, *n* (%)**				
B symptoms	14 (14.3)	26 (34.2)	24 (46.2)	<0.001
Splenomegaly	18 (18.4)	28 (36.8)	26 (50.0)	<0.001
Treatment required	28 (28.6)	40 (52.6)	36 (69.2)	<0.001

SD: Standard deviation. Continuous variables were compared using one-way ANOVA or the Kruskal–Wallis test as appropriate; categorical variables were compared using the chi-square test.

**Table 3:** Comparison of patients with and without treatment requirement.

Variable	No Treatment Required (*n* = 122)	Treatment Required (*n* = 104)	*p*-Value
Age (years), mean ± SD	59.2 ± 13.5	66.1 ± 13.4	<0.001
Hemoglobin (g/dL), mean ± SD	12.3 ± 1.8	9.9 ± 2.0	<0.001
White blood cells (×10^9^/L), mean ± SD	15.1 ± 10.2	22.7 ± 13.4	<0.001
Absolute lymphocyte count (×10^9^/L), mean ± SD	10.2 ± 8.1	17.4 ± 12.1	<0.001
Platelet (×10^9^/L), mean ± SD	192 ± 74	138 ± 86	<0.001
LDH (U/L), median (IQR)	320 (240–460)	540 (380–780)	<0.001
**Clinical Findings, *n* (%)**			
B symptoms	18 (14.8)	46 (44.2)	<0.001
Splenomegaly	26 (21.3)	46 (44.2)	<0.001
High-stage disease	12 (9.8)	40 (38.5)	<0.001

SD: Standard deviation; IQR: Interquartile range; LDH: Lactate dehydrogenase. Continuous variables were compared using an independent samples *t*-test or a Mann–Whitney U test as appropriate; categorical variables were compared using the chi-square test.

**Table 4:** Comparison of patients with and without disease progression.

Variable	No Progression (*n* = 138)	Progression (*n* = 88)	*p*-Value
Age (years), mean ± SD	60.1 ± 13.7	66.0 ± 13.5	0.002
Hemoglobin (g/dL), mean ± SD	12.1 ± 1.9	9.8 ± 2.1	<0.001
White blood cells (×10^9^/L), mean ± SD	15.8 ± 10.9	22.4 ± 13.8	<0.001
Absolute lymphocyte count (×10^9^/L), mean ± SD	11.0 ± 8.9	18.2 ± 12.4	<0.001
Platelet (×10^9^/L), mean ± SD	186 ± 76	132 ± 88	<0.001
LDH (U/L), median (IQR)	340 (260–500)	560 (400–820)	<0.001
**Clinical Findings, *n* (%)**			
B symptoms	20 (14.5)	44 (50.0)	<0.001
Splenomegaly	30 (21.7)	42 (47.7)	<0.001
High-stage disease	14 (10.1)	38 (43.2)	<0.001
Treatment required	48 (34.8)	56 (63.6)	<0.001

SD: Standard deviation; IQR: Interquartile range; LDH: Lactate dehydrogenase. Continuous variables were compared using an independent samples *t*-test or a Mann–Whitney U test as appropriate; categorical variables were compared using the chi-square test.

**Table 5:** Univariate logistic regression analysis for predicting treatment requirement.

Variable	OR	95% CI	*p*-Value
Age (per year)	1.04	1.02–1.06	<0.001
Male sex	1.15	0.68–1.95	0.59
Hemoglobin (per g/dL)	0.72	0.64–0.81	<0.001
White blood cells (per ×10^9^/L)	1.05	1.03–1.07	<0.001
Absolute lymphocyte count (per ×10^9^/L)	1.06	1.03–1.08	<0.001
Platelet (per ×10^9^/L)	0.98	0.97–0.99	<0.001
LDH (per 100 U/L increase)	1.22	1.14–1.31	<0.001
Beta-2 microglobulin (per mg/L)	1.35	1.18–1.55	<0.001
B symptoms (yes vs. no)	4.65	2.50–8.66	<0.001
Splenomegaly (yes vs. no)	2.95	1.65–5.28	<0.001
High-stage disease (vs. low/intermediate)	5.78	2.90–11.52	<0.001

OR: Odds ratio; CI: Confidence interval.

**Table 6:** Multivariable logistic regression analysis for predicting treatment requirement.

Variable	Adjusted OR	95% CI	*p*-Value
Age (per year)	1.03	1.01–1.05	0.004
Hemoglobin (per g/dL)	0.78	0.69–0.89	<0.001
Absolute lymphocyte count (per ×10^9^/L)	1.04	1.02–1.07	<0.001
Platelet (per ×10^9^/L)	0.99	0.98–0.99	0.002
LDH (per 100 U/L increase)	1.16	1.08–1.25	<0.001
B symptoms (yes vs. no)	3.12	1.55–6.28	0.001
Splenomegaly (yes vs. no)	2.21	1.18–4.14	0.013
High-stage disease (vs. low/intermediate)	3.85	1.85–8.02	<0.001

OR: Odds ratio; CI: Confidence interval.

**Table 7:** Hierarchical mixed-effects logistic regression analysis for predicting treatment requirement.

Variable	Adjusted OR	95% CI	*p*-Value
Age (per year)	1.03	1.01–1.05	0.006
Hemoglobin (per g/dL)	0.79	0.70–0.90	<0.001
Absolute lymphocyte count (per ×10^9^/L)	1.04	1.02–1.07	<0.001
Platelet (per ×10^9^/L)	0.99	0.98–0.99	0.003
LDH (per 100 U/L increase)	1.15	1.07–1.24	<0.001
B symptoms (yes vs. no)	2.98	1.48–6.02	0.002
Splenomegaly (yes vs. no)	2.10	1.12–3.95	0.021
High-stage disease (vs. low/intermediate)	3.62	1.72–7.63	<0.001

OR: Odds ratio; CI: Confidence interval.

**Table 8:** Random effects and model fit statistics of the hierarchical model.

Parameter	Value
Diagnostic period variance	0.18
Intraclass correlation coefficient (ICC)	0.05
AIC	214.6
BIC	238.9

ICC: Intraclass correlation coefficient; AIC: Akaike information criterion; BIC: Bayesian information criterion.

**Table 9:** ROC analysis of laboratory parameters and clinical risk score for predicting treatment requirement.

Variable	AUC	95% CI	Cut-Off	Sensitivity (%)	Specificity (%)	*p*-Value
Hemoglobin	0.74	0.68–0.80	≤10.5	72.1	68.0	<0.001
Absolute lymphocyte count	0.71	0.65–0.77	≥15.0	69.2	66.4	<0.001
Platelet	0.69	0.63–0.75	≤140	65.4	64.8	<0.001
LDH	0.78	0.72–0.83	≥450	75.0	70.5	<0.001
Clinical risk score	0.84	0.79–0.89	≥4	81.7	73.8	<0.001

ROC: Receiver operating characteristic; AUC: Area under the curve; CI: Confidence interval. Cut-off values were determined using the Youden index.

**Table 10:** Distribution of clinical outcomes according to risk score categories.

Risk Group	*n*	Treatment Required, *n* (%)	Progression, *n* (%)	Death, *n* (%)	*p*-Value
Low risk	80	12 (15.0)	10 (12.5)	4 (5.0)	<0.001
Intermediate risk	92	42 (45.7)	34 (37.0)	14 (15.2)	<0.001
High risk	54	50 (92.6)	44 (81.5)	24 (44.4)	<0.001

Comparisons between groups were performed using the chi-square test.

## Data Availability

The raw data supporting the conclusions of this article will be made available by the authors on request.

## Abbreviations

The following abbreviations are used in this manuscript:

CLL	Chronic lymphocytic leukemia
ROC	Receiver operating characteristic
AUC	Area under the curve
CI	Confidence interval
OR	Odds ratio
LDH	Lactate dehydrogenase
SD	Standard deviation
IQR	Interquartile range
WBC	White blood cell
ICC	Intraclass correlation coefficient
AIC	Akaike information criterion
BIC	Bayesian information criterion
